# Enhanced Recovery After Surgery Compliance and Outcomes for Head and Neck Reconstructive Surgery

**DOI:** 10.1001/jamaoto.2024.5393

**Published:** 2025-02-27

**Authors:** Chad W. Wagoner, Abby Thomas, Joseph C. Dort, Gregg Nelson, Khara M. Sauro

**Affiliations:** 1 Department of Kinesiology, Recreation, and Sport Studies, University of Tennessee–Knoxville; 2 Department of Community Health Sciences, Cumming School of Medicine, University of Calgary, Calgary, Alberta, Canada; 3 Ohlson Research Initiative, Arnie Charbonneau Research Institute, Cumming School of Medicine, University of Calgary, Calgary, Alberta, Canada; 4 Department of Surgery, Cumming School of Medicine, University of Calgary, Calgary, Alberta, Canada; 5 Department of Oncology, Cumming School of Medicine, University of Calgary, Calgary, Alberta, Canada; 6 O’Brien Institute of Public Health, Cumming School of Medicine, University of Calgary, Calgary, Alberta, Canada; 7 Ariadne Labs, Brigham and Women’s Hospital, Harvard T.H. Chan School of Public Health, Boston, Massachusetts

## Abstract

**Importance:**

Few studies have examined the association between enhanced recovery after surgery (ERAS) compliance and postoperative outcomes within head and neck (HN) free flap reconstructive surgery. Doing so may inform future interventions to improve ERAS adoption and improve postoperative outcomes.

**Objective:**

To assess overall compliance with ERAS guidelines and its association with postoperative outcomes among individuals undergoing HN free flap reconstructive surgery.

**Design, Setting, and Participants:**

This retrospective cohort study included patients who underwent ERAS-guided HN major reconstructive surgery in Alberta, Canada between January 2017 and September 2021. Data analysis occurred from May 2024 until August 2024.

**Main Outcomes and Measures:**

ERAS compliance was assessed for 17 ERAS care elements, and the total compliance score for each patient was a sum of the compliance for each ERAS care element. Compliance was categorized as low compliance (<53%), moderate compliance (53%-72%), and high compliance (>72%). Postoperative outcomes included hospital length of stay and hospital readmission and emergency department admissions within 30 days, intensive care unit readmission, complications, and severe complications. Unadjusted and adjusted models (using backward stepwise regression) assessed associations between ERAS compliance (exposure) and postoperative outcomes.

**Results:**

Of 257 patients, 90 (35.0%) were female, and the mean (SD) age was 62.4 (13.3) years. Overall, 196 (76.3%) had moderate compliance, 50 (19.5%) had low ERAS compliance, and 11 (4.3%) had high compliance. Preoperative (86%) and intraoperative (73%) ERAS compliance exceeded postoperative compliance (38%). Compliance for ERAS care elements varied widely, with the highest compliance observed for preincision antibiotic prophylaxis (99.6%) and the lowest compliance observed for postoperative early mobilization (10.2%). Postoperative hospital length of stay decreased by 0.71 days (95% CI, −1.34 to −0.08), and the odds of experiencing complications decreased by 28% (odds ratio, 0.72; 95% CI, 0.56-0.90) for each 1-unit increase in the total ERAS compliance score.

**Conclusions and Relevance:**

This results of this cohort study suggest that higher overall compliance with ERAS guidelines was associated with improved postoperative outcomes for individuals undergoing major HN free flap reconstructive surgery. There also appeared to be discrepancies in compliance between preoperative and postoperative phases, suggesting areas for interventions designed to improve adherence to ERAS protocols and underscoring the need for proactive compliance monitoring for optimizing patient outcomes in major HN surgery.

## Key Points

**Question:**

What is the association between overall enhanced recovery after surgery (ERAS) compliance and postoperative outcomes among individuals undergoing head and neck (HN) free flap reconstructive surgery?

**Findings:**

In this cohort study of 257 patients who underwent HN free flap reconstructive surgery, higher overall compliance with ERAS guidelines was associated with improved postoperative outcomes, but there were discrepancies in compliance between preoperative and postoperative phases.

**Meaning:**

The results of this study support the use of ERAS guidelines for improving postoperative outcomes after HN free flap reconstructive surgery and highlight the need for targeted interventions that are associated with improved ERAS compliance across operative phases, which may optimize outcomes in major HN surgery.

## Teaser

This cohort study examines the association between compliance with enhanced recovery after surgery guidelines and postoperative outcomes among individuals undergoing head and neck free flap reconstructive surgery.

## Introduction

Head and neck (HN) cancers are the sixth most common cancer worldwide, with an estimated 890 000 new cases diagnosed annually.^1,2^ One of the most common primary treatments is surgical resection of the tumor with free flap reconstruction. Postoperative complications after major HN surgery may include pneumonia, sepsis, and infection at the surgical site,^3^ which are associated with greater length of hospital stay^3^ and are burdensome for patients and the health care system.^4^ To improve postoperative outcomes, an enhanced recovery after surgery (ERAS) guideline was published in 2017 for major HN surgery with free flap reconstruction.^5^ ERAS guidelines were originally developed for colorectal cancer^6^ and were subsequently adapted for other surgical procedurers.^7^ These guidelines include numerous care elements across preoperative, intraoperative, and postoperative surgical phases. Across surgeries, including major HN surgery, implementing ERAS guidelines have been associated with decreased complications, hospital and intensive care unit (ICU) length of stays, and health care costs and may be associated with an improved return to intended oncologic treatment.^8,9,10,11,12,13,14^

There is evidence to suggest that better compliance with ERAS guidelines (ie, the proportion of individuals with adherence to all ERAS care elements) is associated with better postoperative outcomes, such as decreased hospital length of stay, after major surgery.^15,16^ However, a recent meta-analysis found that few studies have looked at the association of ERAS compliance with postoperative outcomes within HN free flap reconstructive surgery cohorts.^17^ Measuring overall ERAS compliance and identifying ERAS care elements with poor compliance may inform future interventions to improve ERAS adoption, which may be associated with better postoperative outcomes. Therefore, the purpose of this study was to determine overall compliance with ERAS HN guidelines and its association with postoperative outcomes for those who have undergone major HN surgery with free flap reconstruction.

## Methods

### Setting

This study was conducted in Alberta, Canada, which has a publicly funded health care system in which a single health care network (Alberta Health Services) delivers health care services to the entire province. All ERAS-guided HN free flap reconstructive surgeries in Southern Alberta were performed at the Foothills Medical Centre in Calgary, Alberta, Canada, between January 2017 and September 2021. This study was approved by the Conjoint Health Research Ethics Board at the University of Calgary (Alberta, Canada), which waived patient consent.

### Study Design and Participants

This retrospective cohort study included adult patients (age ≥18 years) who underwent ERAS-guided HN ablative/free flap reconstructive surgery. The cohort was identified using the ERAS Interactive Audit System (EIAS), a dataset of all patients who underwent an ERAS-guided procedure in Alberta. Patients for whom the ERAS guidelines were not applicable (eg, wrong type of surgery) were excluded. The study followed the Strengthening the Reporting of Observational Studies in Epidemiology (STROBE) reporting guideline.

### Data Sources

The EIAS, which collects information about patient demographic characteristics, health status, surgical details, complications, and ERAS compliance across all surgical types,^18,19^ was linked to 3 population-based, routinely collected health databases to gain more information about the hospital stay and patients’ interactions with the health care system after hospital discharge. Using the *International Statistical Classification of Diseases and Related Health Problems, Tenth Revision * Canadian codes (*ICD-10-CA*), the Discharge Abstract Database (DAD) contains up to 25 diagnostic codes and includes information on demographic characteristics, administrative data, and procedural data from all patients discharged from the hospital.^20^ The DAD was used to collect information on hospital length of stay, readmissions, and postoperative complications. The National Ambulatory Care Reporting System (NACRS) collects clinical, administrative, and service-specific data from hospital and community-based ambulatory visits.^21^ For this study, NACRS was used to determine if participants had visited an emergency department (ED) within 30 days of hospital discharge. eCritical TRACER, which is an electronic medical record and database that provides multimodal patient data, such as clinical and demographic data, provided information associated with postoperative ICU readmission.^22^

### Cohort Characteristics Variables

Demographic characteristics were abstracted for each patient and included age (continuous, years), sex (dichotomous, female or male), and location of residence (dichotomous, rural or urban). Socioeconomic status was derived using the Pampalon Deprivation Index^23^ and consisted of 2 components: material deprivation (categorical, 1 = least material deprivation to 5 = most material deprivation) and social deprivation (categorical, 1 = least social deprivation to 5 = most social deprivation). Material deprivation reflects the level of deprivation of wealth and goods, whereas social deprivation reflects the level of deprivation in family and community relationships. Patient operative risk (dichotomous, low risk or high risk) was determined by the American Society of Anesthesiologists physical status score. To determine the number of comorbidities, the Charlson Comorbidity Index score was calculated using the Quan algorithm based on *ICD-10* codes.^24^ These continuous scores were then categorized for analysis (categorical, 0 = no comorbidities; 1 = 1 comorbidity; ≥2 = 2 or more comorbidities).

### Primary Exposure Outcome: ERAS Compliance

ERAS guidelines included 17 care elements across preoperative, intraoperative, and postoperative free flap reconstruction surgical phases, which included the following: nutrition guidelines, patient education guidelines, alcohol use guidelines, smoking recommendations, oral carbohydrate recommendations, long sedation guidelines, thrombosis prophylaxis, preincision antibiotic prophylaxis, postoperative nausea and vomiting prophylaxis, pain management (long-acting opioid use), intravenous fluid management, forced heating, termination of urinary drainage, termination of intravenous fluid use, closed tracheostomy, early mobilization (day of surgery), and early mobilization. Operational definitions of compliance for each ERAS care element are described in eTable 1 in Supplement 1. All ERAS care elements were categorical and dichotomized as compliant or noncompliant.

A total compliance score for each patient was calculated by summing the number of compliant ERAS care elements. A compliance score could range from 0, representing no compliance, to 17, representing compliance for all care elements. If an ERAS care element status for a specific patient was missing (ie, no indication of compliance or noncompliance), case-wise deletion was used for that analysis rather than assuming a compliance of 0. A categorical compliance level was calculated using the compliance proportion (number of compliant care elements / 17 × 100) to categorize compliance as low compliance (<53%), moderate compliance (53%-71%), and high compliance (>71%). ERAS compliance levels were chosen based on previous work from members of the research team exploring ERAS compliance levels across colorectal, pancreatic, gynecologic, and liver surgery populations.^16^

### Secondary Outcome

Outcomes included hospital length of stay, hospital readmission within 30 days of index discharge, ED visits within 30 days of hospital discharge, ICU readmission, and complications. Hospital length of stay was calculated as the number of days from admission to discharge in the DAD (continuous). Hospital readmission was a dichotomous variable (yes/no) derived from the DAD that indicated if the patient had a hospital readmission within 30 days of the discharge date from the index hospitalization. An ED visit was a dichotomous variable (yes/no) derived from NACRS that indicated if the patient had visited the ED within 30 days of discharge from their surgical hospital stay. ICU readmission (dichotomous, yes/no) was derived from the eCritical database (Alberta Health Services) and was defined as any readmission to the ICU following the initial surgical admission as part of standard care. Complications, which were abstracted from the EIAS, was a dichotomous variable (yes/no) indicating if a patient experienced any type of complication during their hospital stay. Severe complications, which were abstracted from EIAS, was a dichotomous variable (yes/no) indicating whether the complications experienced were severe. Severity was determined using the Clavien Dindo Scale and categorized into no severe complications (grade I-II) and severe complications (grade IIIa-V).^16,25^

### Statistical Analysis

All data were analyzed using R-Studio, version 4.3.0 (R Foundation). Descriptive statistics summarized the demographic characteristics of the cohort, compliance with individual ERAS care elements, and primary outcome variables using frequencies (proportions), means (SDs) and medians (IQRs) as appropriate. Demographic characteristics of the cohort were also stratified by compliance level. Effect sizes were calculated to evaluate the difference in cohort characteristics and outcomes across ERAS compliance levels (η^2^ for continuous variables and Kendall τ for categorical variables). A sensitivity analysis with different compliance threshold levels (0%-32%, 33%-66%, and 67%-100% compliance) was conducted to ensure the robustness of the results (eTable 2 in Supplement 1). Case-wise deletion was implemented for each model to handle missing data. Unadjusted univariable models were used to determine the association between the outcomes and the total ERAS compliance score (exposure). Adjusted multivariable models with backward stepwise regression were used to determine the same associations while considering demographic characteristics (age, sex, and operative risk). We chose these variables in consultation with clinical experts, which suggests that these factors may be associated with ERAS compliance.

## Results

### Cohort Characteristics

The cohort included 257 patients with complete ERAS data who underwent major HN surgery with free flap reconstruction. Patients who had incomplete ERAS data (52 [16.8%]) were not analyzed. Patients in the cohort were had a mean (SD) age of 62.4 (13.3) years, were predominantly male (167 [65.0%]), primarily resided in urban locations (203 [79.0%]), and had no comorbid conditions per the Charlson Comorbidity Index score (170 [66.1%]).

### ERAS Compliance

The mean overall ERAS compliance was 62.6%, ranging from 10.2% to 99.6% across all ERAS elements. Compliance was low among 50 patients (19.5%), moderate among 196 patients (76.3%), and high among 11 patients (4.3%). There were no differences in characteristics between patients with low, moderate, and high compliance (Table 1; eTable 2 in Supplement 1).

**Table 1.** Cohort Characteristics^a^

Variables	No. (%)				Effect size (95% CI)
	Total (N = 257)	Compliance			
		Low (n = 50)	Moderate (n = 196)	High (n = 11)	
Age, mean (SD), y	62.4 (13.3)	61.7 (13.5)	62.7 (13.3)	60.0 (14.7)	0.002 (0 to 0.020)
Sex					
Female	90 (35.0)	17 (18.9)	69 (76.7)	4 (4.4)	−0.011
Male	167 (65.0)	33 (19.8)	127 (76.0)	7 (4.2)	
Location of residence					
Rural	46 (17.9)	12 (26.1)	31 (67.4)	3 (6.5)	−0.038
Urban	203 (79.0)	38 (18.7)	159 (78.3)	6 (3.0)	
No information provided	8 (3.1)	0	6 (75.0)	2 (25.0)	
Material deprivation					
Least deprivation	48 (18.7)	10 (20.8)	38 (79.2)	0	0.035
2	43 (16.7)	9 (20.9)	34 (79.1)	0	
3	50 (19.5)	9 (18.0)	39 (78.0)	2 (4.0)	
4	46 (17.8)	7 (15.2)	37 (80.4)	2 (4.4)	
Most deprivation	58 (22.6)	14 (24.1)	40 (70.0)	4 (6.9)	
No information provided	12 (4.7)	1 (8.3)	8 (66.7)	3 (25.0)	
Social deprivation					
Least deprivation	42 (16.3)	9 (21.4)	32 (76.2)	1 (2.4)	0.022
2	48 (18.7)	10 (20.8)	37 (77.1)	1 (2.1)	
3	45 (17.5)	10 (22.2)	33 (73.3)	2 (4.4)	
4	50 (19.5)	7 (14.0)	41 (82.0)	2 (4.0)	
Most deprivation	60 (23.3)	13 (21.7)	45 (75.0)	2 (3.3)	
No information provided	12 (4.7)	1 (8.3)	8 (66.7)	3 (25.0)	
Operative risk (American Society of Anesthesiologists)					
Lower risk (1 and 2)	138 (53.7)	24 (17.4)	107 (77.5)	7 (5.1)	−0.066
Higher risk (3 and 4)	118 (45.9)	26 (22.0)	88 (74.6)	4 (3.4)	
No information provided	1 (0.4)	0	1 (100.0)	0	
Charlson Comorbidity Index score					
0 Comorbid conditions	170 (66.1)	33 (19.4)	131 (77.1)	6 (3.5)	0.024
1 Comorbid condition	13 (5.1)	3 (23.1)	10 (76.9)	0	
≥2 Comorbid conditions	74 (28.8)	14 (18.9)	55 (74.3)	5 (6.8)	

^a^ No significant differences were observed between groups. η^2^ reported for continuous variables; Kendall τ for categorical variables.

Compliance varied for each ERAS care element. The preoperative phase had the highest ERAS compliance (86.0% compliance), whereas the postoperative phase had the lowest compliance (38.3% compliance range, 10.2%-86.8%), with mobilization on the day of surgery (10.2% compliance) and early mobilization within 24 hours of surgery (17.5% compliance) having the lowest compliance. The mean intraoperative ERAS compliance was 73.1% and ranged from 38.1% to 99.2%. Compliance for each individual ERAS care element can be seen in Table 2.

**Table 2.** Individual Enhanced Recovery After Surgery (ERAS) Care Element Compliance

ERAS care element	Total No.	Individuals with compliance, No. (%)
Preoperative compliance		
Preincision antibiotic prophylaxis	257	256 (99.6)
Thrombosis prophylaxis	255	243 (95.3)
Nutrition guidelines	256	241 (94.1)
Patient education guidelines	247	231 (93.5)
Long sedation guidelines	256	227 (88.7)
Postoperative nausea and vomiting prophylaxis	255	208 (81.6)
Oral carbohydrate recommendations	250	193 (77.2)
Alcohol use guidelines	245	189 (77.1)
Smoking recommendations	85	10 (11.8)
Intraoperative compliance		
Forced heating	253	251 (99.2)
Intravenous fluid management	257	211 (82.1)
Pain management	257	98 (38.1)
Postoperative compliance		
Trachea closed	159	138 (86.8)
Termination of urinary drainage	253	161 (63.6)
Early mobilization	257	45 (17.5)
Termination of intravenous fluid use	250	33 (13.2)
Early mobilization day of surgery	236	24 (10.2)

### Association Between Outcomes and Compliance Level

Postoperative outcomes were stratified and reported by compliance level (Table 3). Between-group tests of significance were not conducted, and only descriptive statistics were reported (mean [SD] or number [%]) due to the small number of patients in the low and high compliance categories). Postoperative length of hospital stay ranged from a mean (SD) of 10.9 (7.1) days (high compliance) to 12.5 (6.6) days (low compliance), readmission to the ICU ranged from 36.4% (high compliance: 4 of 11) to 48.0% (low compliance: 22 of 50), and experiencing complications ranged from 63.6% (high compliance: 7 of 11) to 84.0% (low compliance: 42 of 50). Descriptive statistics of postoperative outcomes for each individual ERAS care element were also derived and can be seen in eTable 3 in Supplement 1.

**Table 3.** Postoperative Outcomes Stratified by Enhanced Recovery After Surgery Compliance Level^a^

Clinical outcome	No. (%)			Effect size (95% CI)
	Compliance			
	Low (n = 50)	Moderate (n = 196)	High (n = 11)	
Postoperative hospital length of stay, mean (SD), d	12.5 (6.6)	11.1 (7.6)	10.9 (7.1)	0.006 (0 to 0.032)
Readmitted to hospital <30, d	6 (12.0)	11 (5.6)	1 (9.1)	0.077
Visited ED <30, d	4 (8.0)	8 (4.1)	0	−0.085
Readmitted to ICU	24 (48.0)	94 (48.0)	4 (36.4)	0.083
Experienced any complications	42 (84.0)	146 (74.5)	7 (63.6)	−0.102
Experienced any severe complications	26 (52.0)	98 (50.0)	6 (54.5)	−0.006

Abbreviations: ED, emergency department; ICU, intensive care unit.

^a^ η^2^ Reported for continuous variables; Kendall τ for categorical variables.

Unadjusted models showed that postoperative hospital length of stay decreased by 0.71 days (95% CI, −1.34 to −0.08), and the odds of experiencing complications decreased by 28% (odds ratio [OR], 0.72; 95% CI, 0.56-0.90) for each 1-unit increase in the total ERAS compliance score. Being readmitted to the hospital (OR, 0.89; 95% CI, 0.65-1.23), being readmitted to the ICU (OR, 0.88; 95% CI, 0.74-1.04), visiting the ED within 30 days of discharge (OR, 0.90; 95% CI, 0.62-1.35), or experiencing severe complications (OR, 1.03; 95% CI, 0.87-1.22) were not associated with the total ERAS compliance score. After using backwards elimination, sex, age, and operative risk were not found to modify or confound the association between the outcomes and compliance; therefore, only unadjusted estimates are presented (Figure). eTable 4 in Supplement 1 describes the unadjusted and adjusted model results.

**Figure.**
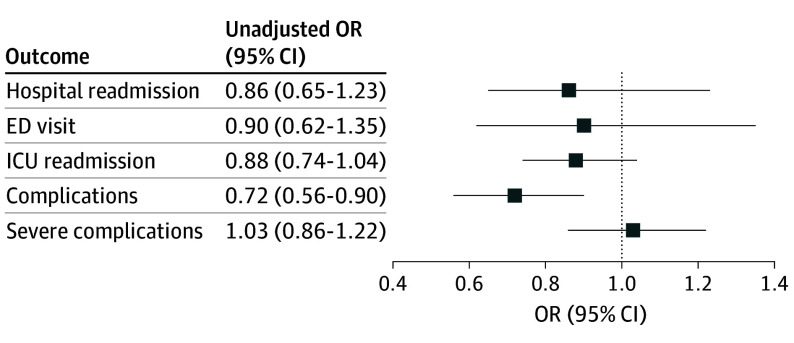
Postoperative Outcomes of Enhanced Recovery After Surgery Compliance ED indicates emergency indicates; ICU, intensive care unity; OR, odds ratio.

## Discussion

The results of this cohort study suggest that overall compliance was moderate (62.6%). Preoperative (86% compliance) and intraoperative ERAS compliance (73% compliance) was substantially greater than postoperative ERAS compliance (38% compliance) among patients undergoing major HN surgery with free flap reconstruction. Also, compliance varied by ERAS care element. The lowest compliance was reported for early mobilization during the postoperative phase (10.2%), and the greatest compliance was reported for preincision antibiotic prophylaxis (99.6%). Compliance was associated with better postoperative outcomes, including reduced hospital length of stay and odds of experiencing complications.

Previous studies have shown that implementing ERAS guidelines associated with better postoperative outcomes across surgical disciplines, including reduced hospital length of stay and complications.^7,9,10,11,12,13,15,26^ Similar to the present study, the greater ERAS compliance derived from EIAS has been shown to be associated with reduced hospital length of stay across populations with pancreatic, liver, colorectal, and urologic disease.^27^ However, to our knowledge, ERAS compliance and its association with postoperative outcomes has not been studied extensively in HN surgery. For example, a recent study in HN surgery reported that increased ERAS compliance was associated with reduced hospital length of stay.^28^ These findings were limited to nutritional compliance only. The present study builds on this work and addresses this knowledge gap by reporting on ERAS compliance across multiple care elements for major HN surgery with free flap reconstruction and evaluating their association with multiple postoperative outcomes. The potential association ERAS compliance has with health care costs suggests that improving overall compliance to ERAS guidelines should be prioritized for individuals undergoing major HN surgery with free flap reconstruction.^7,15^ For example, financial savings have been associated with greater ERAS compliance when it is derived from EIAS, although this is not specific to HN surgery.^27^ Although our study was not focused on health care costs, it is reasonable to consider that the reductions in hospital length of stay and complications associated with better ERAS compliance observed in this study may also be associated with reduced health care costs for this patient population.

Although overall ERAS compliance was associated with better postoperative outcomes after major HN surgery with free flap reconstruction, it remains unclear whether compliance with individual ERAS care elements is associated with postoperative outcomes more or less than others. Although our study was not designed to address this question, it was clear that postoperative compliance was lower than preoperative and intraoperative compliance for individual ERAS care elements, which was consistent with previous work in colorectal surgery.^15^ Although this study did not test the association between postoperative outcomes with individual ERAS care elements compliance due to it requiring multiple hypothesis testing, trends were observed between individual ERAS care element compliance and postoperative outcomes (eTable 2 in Supplement 1). In particular, early mobilization on postoperative day 0 and days 1 to 3 reported some of the lowest compliance (10.2% and 17.5% compliance, respectively). This coincided with greater hospital length of stays as well as greater percentages for being readmitted to the ICU and experiencing complications for those who with noncompliance. These trends were supported by previous work from our group that found as association of early mobilization within 48 hours after HN free flap reconstructive surgery with reduced postoperative complications.^29^ These findings suggest that future work should explore the variation of ERAS compliance across individual care elements and their relative association with postoperative outcomes. Exploring this research question may provide insight into the relative importance of individual ERAS care elements and postoperative outcomes.

ERAS implementation and high compliance are associated with improved postoperative outcomes^7,9,10,11,12,13,15,26^ and underscore a need to design interventions that will improve ERAS compliance. By tracking compliance for individual ERAS care elements, this study identified targets most likely to be improved by novel interventions. Intravenous fluid use or early mobilization during the postoperative phase are examples of ERAS care elements worthy of focused attention. Although tracked as a postoperative ERAS care element, termination of intravenous fluid use depends on preoperative and intraoperative fluid management, during which excessive fluid use may be associated with postoperative complications.^30^ The findings from this study suggest that interventions for improved fluid management during the preoperative and intraoperative phases may be necessary to improve postoperative intravenous fluid use compliance. Goal-directed fluid therapy (ie, administering fluids based hemodynamic criteria) is a promising intervention within HN cancer surgery that has been associated with better postoperative outcomes.^31^ Future interventions may consider exploring whether goal-directed fluid therapy during the intraoperative phase is associated with termination of intravenous fluid use during the postoperative phase for major HN surgery with free flap reconstruction.

To address low early mobilization compliance, our group recently implemented a multiphasic exercise prehabilitation intervention before HN cancer free flap reconstructive surgery that includes exercise and early mobilization education during the preoperative phase.^32^ The intervention was based on previous prehabilitation interventions in breast cancer and colorectal surgery were associated with improved physical function^33^ and reduced hospital length of stay.^34^ This prehabilitation intervention was designed to improve early mobilization compliance among HN cancer surgery patients by addressing its physical demands through exercise and providing education on what early mobilization entails and the benefits it provides. Patients and health care clinicians saw value in implementing exercise prehabilitation programs before HN cancer free flap reconstructive surgery.^35^ Thus, this work may serve as an example of how to address low ERAS compliance during the postoperative phase by intervening during the preoperative phase and improving postoperative outcomes.

### Limitations

Limitations of this study should be noted. First, this was a retrospective cohort study design. Thus, despite seeing associations between high ERAS compliance and better postoperative outcomes, a causal relationship cannot be determined, and our results should be interpreted with this in mind. The sample was also subject to inconsistent ERAS compliance reporting. For example, there were substantial missing data on compliance with smoking recommendations, which is concerning given the potential adverse association smoking may have with postoperative recovery. As a result, analyses appropriate for testing the association of individual ERAS care element compliance with postoperative outcomes were not conducted. Lastly, clinically relevant variables that could unpack the complex association between treatment-related variables among patients with HN cancers (who account for most HN surgeries) were not available in our dataset (eg, HN subsite, tumor stage, and node stage). Future prospective studies should consider including additional oncologic variables, such as those described previously, and examine the association between ERAS compliance and return to intended oncologic treatment,^14^ such as time to adjuvant therapy. Despite these limitations, a strength of this study was that the findings may inform future research that explores ERAS compliance, implementation, and the relative association of individual ERAS care elements with clinical outcomes. Specifically, future studies should not only focus on the effects of pre-/post- ERAS implementation, but also on reporting compliance for each ERAS care element. Doing so may provide more personalized approaches to enhanced recovery using focused analytic approaches that examine the overall and component-specific association of ERAS compliance with postoperative outcomes that may be followed by improved ERAS implementation for patients undergoing surgery.

## Conclusions

The results of this cohort study suggest that higher total ERAS compliance was associated with better postoperative outcomes. These findings were consistent with previous studies that focused on the effects of ERAS implementation in HN cancer and other surgical procedures and builds on them by also considering the association of ERAS compliance with postoperative outcomes for major HN surgery with free flap reconstruction. Our results also identified a need to focus on improving compliance with postoperative ERAS care elements. Future research focusing on the relative association of individual ERAS care elements with clinical outcomes would be especially valuable to streamline ERAS protocols and make them accessible to a broad range of clinical programs.

## Notes

Supplement 1. eTable 1. Operation Definitions of ERAS Components eTable 2. Sensitivity Analysis eTable 3. Proportion of Post-Operative Outcome Occurrence with Individual ERAS Components eTable 4. Unadjusted and Adjusted Model Results

Supplement 2. Data sharing statement
