# Supplementary material for: Enhanced Recovery After Surgery Compliance and Outcomes for Head and Neck Reconstructive Surgery
Source: JAMA Otolaryngol Head Neck Surg. 2025 Feb 27;151(4):371–8. doi: 10.1001/jamaoto.2024.5393 (PMC11869090; doi:10.1001/jamaoto.2024.5393)
Supplement: Supplement 1. — eTable 1. Operation Definitions of ERAS Components eTable 2. Sensitivity Analysis eTable 3. Proportion of Post-Operative Outcome Occurrence with Individual ERAS Components eTable 4. Unadjusted and Adjusted Model Results [file jamaotolaryngolheadnecksurg-e245393-s001.pdf]

Supplemental Online Content

Wagoner CW, Thomas A, Dort JC, Nelson G, Sauro KM. Enhanced recovery after surgery compliance and outcomes for head and neck reconstructive surgery. *JAMA Otolaryngol Head Neck Surg*. Published online February 27, 2025.
doi:10.1001/jamaoto.2024.5393

eTable 1. Operation Definitions of ERAS Components
eTable 2. Sensitivity Analysis
eTable 3. Proportion of Post-Operative Outcome Occurrence with Individual ERAS Components
eTable 4. Unadjusted and Adjusted Model Results

This supplemental material has been provided by the authors to give readers additional information about their work.

© 2025 Wagoner CW et al. *JAMA Otolaryngology–Head & Neck Surgery*.

eTable 1. Operation Definitions of ERAS Components

ERAS Component	Question	Measurement
Pre-Operative Compliance		
Nutrition Guidelines	Binary variable indicating if patients were compliant with the ERAS pre-operative nutrition guidelines.	Compliant = patients who received treatment if they were at risk of malnutrition before surgery and did not receive treatment if they were deemed healthy/normal status.
Patient Education Guidelines	Binary variable indicating if patients received pre-admission education or not.	Compliant = patients who received education about their surgery prior to hospital admission.
Alcohol Usage Guidelines	Binary variable indicating if patients complied with pre-operative alcohol use guidelines.	Compliant = patients who did not drink alcohol at all before surgery, or those who stopped because of surgery.
Smoking Recommendations	Binary variable indicating if patients who smoke complied with the pre-surgery smoking cessation guidelines.	Compliant = patients who stopped smoking 4 weeks or more before their surgery date.
Oral Carbohydrate Recommendations	Binary variable indicating if patients received pre-operative oral carbohydrates or not.	Compliant = patients who received oral carbohydrates before surgery, and those that did not receive oral carbohydrates before surgery because they were contraindicated.
Long Sedation Guidelines	Binary variable indicating if patients received pre-operative long sedation or not.	Compliant = patients who did not receive pre-operative long sedation.
Thrombosis Prophylaxis	Binary variable indicating if patients received thrombosis prophylaxis before surgery, and if so which type they received.	Compliant = patients that received either heparin or low-molecular weight heparin (anticoagulant) or a combination of compression and anticoagulants.

© 2025 Wagoner CW et al. *JAMA Otolaryngology–Head & Neck Surgery*.

Pre-Incision Antibiotic Prophylaxis	Binary variable indicating if patients received antibiotic prophylaxis before the incision was made.	Compliant = patients that received either IV or oral antibiotics before incision.
PONV Prophylaxis	Binary variable indicating if patients received PONV prophylaxis before surgery.	Compliant patients were those that received PONV prophylaxis before surgery.
Intra-Operative Compliance		
Pain Management	Binary variable indicating if patients received long-acting opioids.	Compliant = patients that did not receive long-acting opioids.
Intravenous Fluid Management	Binary variable indicating if patients received less than 5000 ml of intraoperative fluid.	Compliant = patients that received less than 5000 ml of intraoperative fluid.
Forced Heating	Binary variable indicating if patients received forced heating during surgery.	Compliant = patients that received forced heating during surgery.
Post-Operative Compliance		
Termination of Urinary Drainage	Binary variable indicating if patients were compliant with the guidelines surrounding the timing of the termination of urinary drainage after surgery.	Compliant = patients whose urinary drainage was terminated within 48 hours of the end of their operation.
Termination of Intravenous Fluid Use	Binary variable indicating if patients were compliant with the guidelines surrounding the cessation of IV fluid use after surgery.	Compliant = patients whose IV fluid use stopped within 24 hours of the end of surgery.

© 2025 Wagoner CW et al. *JAMA Otolaryngology–Head & Neck Surgery*.

Trach Closed	Binary variable indicating if patients' trach was closed post-operatively.	Compliant = patients whose trach was closed post-operatively.
Early Mobilization Day of Surgery	Binary variable indicating if patients were mobilized early post-operatively the day of surgery.	Compliant = patients who were mobilized the day of surgery.
Early Mobilization	Binary variable indicating if patients were mobilized early post-operatively.	Compliant = patients who were mobilized within 24 hours of surgery.

© 2025 Wagoner CW et al. *JAMA Otolaryngology–Head & Neck Surgery*.

eTable 2. Sensitivity Analysis

	Mean (SD) or Number (%)				
**Variables**	Total (n=257)	0 to 32% Compliance (n=0)	33 – 66% Compliance (n=176)	67 – 100% Compliance (n=81)	Effect size (95% CI)
Age	62.4 (13.3)		62.6 (13.3)	61.8 (13.5)	0.001 (0.000 – 0.021)
Sex					-0.0989
Female	90 (35.0)		56 (62.2)	34 (37.8)	
Male	167 (65.0)		120 (71.9)	47 (28.1)	
Location of Residence					0.1247
Rural	46 (17.9)		26 (56.5)	20 (43.5)	
Urban	203 (79.0)		145 (71.4)	58 (28.6)	
No information provided	8 (3.1)		5 (62.5)	3 (37.5)	
Material Deprivation					0.0034
Least deprived	48 (18.7)		33 (68.8)	15 (31.3)	
2	43 (16.7)		31 (72.1)	12 (27.9)	
3	50 (19.5)		33 (66.0)	17 (34.0)	
4	46 (17.8)		30 (65.2)	16 (34.8)	
Most deprived	58 (22.6)		41 (70.7)	17 (29.3)	
No information provided	12 (4.7)		8 (66.7)	4 (33.3)	
Social Deprivation					-0.0326
Least deprived	42 (16.3)		26 (61.9)	16 (38.1)	
2	48 (18.7)		33 (68.8)	15 (31.3)	
3	45 (17.5)		35 (77.8)	10 (22.2)	
4	50 (19.5)		31 (62.0)	19 (38.0)	
Most deprived	60 (23.3)		43 (71.7)	17 (28.3)	
No information provided	12 (4.7)		8 (66.7)	4 (33.3)	
Operative Risk (ASA)					-0.0993
Lower risk (1 & 2)	138 (53.7)		89 (64.5)	49 (35.5)	
Higher risk (3 & 4)	118 (45.9)		87 (73.7)	31 (26.3)	
No information provided	1 (0.4)		0 (0.0)	1 (100.0)	
Charlson Comorbidity					-0.0070
0 comorbid conditions	170 (66.1)		116 (68.2)	54 (31.8)	
1 comorbid condition	13 (5.1)		9 (69.2)	4 (30.8)	
2+ comorbid conditions	74 (28.8)		51 (68.9)	23 (31.1)	

Note: no significant differences (p > 0.05) were observed between groups
CI, Confidence Interval
η^2 reported for continuous variables; Kendall's τ for categorical variables.

Post-Operative Outcomes Stratified by ERAS Compliance Level

© 2025 Wagoner CW et al. *JAMA Otolaryngology–Head & Neck Surgery*.

Clinical Outcome	Mean (SD) or Number (%)<br>0 to 32% Compliance (n=0)	33 – 66% Compliance (n=176)	67 – 100% Compliance (n=81)	Effect size (95% CI)
Post-operative Hospital Length of Stay (days)		12.1 (8.2)	9.77 (4.9)	0.0214 (0.001 – 0.069)
Readmitted to Hospital (< 30 days)		12 (6.8)	6 (7.4)	-0.0107
Visited ED (< 30 days)		9 (5.1)	3 (3.7)	-0.0310
Admitted to ICU		80 (71.4)	32 (28.6)	-0.0557
Experienced Any Complications		143 (73.3)	52 (26.7)	-0.1852
Experienced Any Severe Complications		88 (67.7)	42 (32.3)	0.0172

ED, Emergency Department; ICU, Intensive Care Unit; CI, Confidence Interval

η^2 reported for continuous variables; Kendall's τ for categorical variables.

© 2025 Wagoner CW et al. *JAMA Otolaryngology–Head & Neck Surgery*.

eTable 3. Proportion of Post-Operative Outcome Occurrence with Individual ERAS Components

		Clinical Outcome (Average or Number of Occurrences)				
ERAS Component	Hosp. LOS	Readmitted to Hospital	Visited ED	Admitted to ICU	Experienced Complications	Experienced Severe Complications
	Mean (SD)	Number (%)				
Pre-Operative Compliance						
Nutrition Guidelines						
Compliant (n=241)	11.5 (7.5)	17 (7.1)	12 (5.0)	115 (47.7)	184 (76.3)	66 (27.4)
Non-Compliant (n=15)	9.5 (4.2)	1 (6.7)	0 (0.0)	6 (40.0)	10 (66.7)	1 (6.7)
Patient Education Guidelines						
Compliant (n=231)	11.3 (7.5)	14 (6.1)	8 (3.5)	111 (48.1)	173 (74.9)	60 (26.0)
Non-Compliant (n=16)	10.9 (5.8)	2 (12.5)	1 (6.3)	6 (37.5)	14 (87.5)	5 (31.3)
Alcohol Usage Guidelines						
Compliant (n=189)	10.8 (7.4)	13 (6.9)	9 (4.8)	83 (43.9)	137 (72.5)	50 (26.5)
Non-Compliant (n=56)	12.8 (7.4)	3 (5.3)	2 (3.8)	36 (64.3)	46 (82.1)	15 (26.8)
Smoking Recommendations						
Compliant (n=10)	14.5 (7.3)	1 (10.0)	2 (20.0)	6 (60.0)	8 (80.0)	3 (30.0)
Non-Compliant (n=75)	13 (5.8)	1 (20.0)	1 (1.3)	42 (56.0)	5 (100.0)	1 (20.0)
Oral Carb Recommendations						
Compliant (n=193)	11.6 (7.8)	12 (6.2)	9 (4.7)	95 (49.2)	149 (77.2)	52 (26.9)
Non-Compliant (n=57)	10.6 (6.0)	6 (10.5)	3 (5.3)	22 (38.6)	41 (71.9)	15 (26.3)

© 2025 Wagoner CW et al. *JAMA Otolaryngology–Head & Neck Surgery*.

Long Sedation Guidelines						
Compliant (n=227)	11.6 (7.7)	15 (6.6)	11 (4.8)	107 (47.1)	174 (76.7)	61 (26.9)
Non-Compliant (n=29)	9.4 (3.6)	3 (10.3)	1 (3.4)	14 (48.3)	20 (69.0)	7 (24.1)
Thrombosis Prophylaxis						
Compliant (n=243)	11.4 (7.4)	18 (7.4)	12 (4.9)	113 (46.5)	186 (76.5)	64 (26.3)
Non-Compliant (n=12)	9.4 (5.4)	0 (0.0)	0 (0.0)	7 (58.3)	8 (66.7)	3 (25.0)
Pre-Incision Antibiotic Prophylaxis						
Compliant (n=256)	11.4 (7.4)	18 (8.6)	11 (4.3)	122 (47.7)	195 (76.2)	68 (26.6)
Non-Compliant (n=1)	6.0	0 (0.0)	1 (100.0)	0 (0.0)	0 (0.0)	0 (0.0)
PONV Prophylaxis						
Compliant (n=208)	11.5 (7.8)	14 (6.7)	8 (3.8)	94 (45.2)	151 (72.6)	53 (25.5)
Non-Compliant (n=47)	10.7 (5.2)	4 (8.5)	4 (8.5)	27 (57.4)	42 (89.4)	15 (31.9)
Intra-Operative Compliance						
Pain Management						
Compliant (n=98)	10.7 (6.1)	9 (9.1)	3 (3.1)	46 (46.9)	74 (75.5)	48 (49.0)
Non-Compliant (n=159)	11.7 (8.1)	9 (5.7)	9 (5.7)	76 (47.8)	121 (76.1)	82 (51.5)
Intravenous Fluid Management						
Compliant (n=211)	11.0 (7.3)	15 (7.1)	8 (3.8)	95 (45.0)	157 (74.4)	106 (50.2)
Non-Compliant (n=46)	12.8 (7.4)	3 (6.5)	4 (8.7)	27 (58.7)	38 (82.6)	24 (52.2)
Forced Heating						
Compliant (n=251)	11.4 (7.5)	18 (7.2)	11 (4.4)	118 (47.0)	190 (75.7)	64 (25.5)

© 2025 Wagoner CW et al. *JAMA Otolaryngology–Head & Neck Surgery*.

Non-Compliant (n=2)	9.5 (0.7)	0 (0.0)	0 (0.0)	1 (50.0)	1 (50.0)	1 (50.0)
<u>Post-Operative Compliance</u>						
Termination of Urinary Drainage						
Compliant (n=161)	10.3 (6.7)	8 (4.9)	9 (5.6)	75 (46.6)	113 (70.2)	30 (18.6)
Non-Compliant (n=92)	13.3 (8.0)	10 (10.9)	3 (3.3)	47 (51.1)	81 (88.0)	37 (40.2)
Termination of IV						
Compliant (n=33)	5.7 (3.0)	2 (6.1)	3 (9.1)	5 (15.2)	16 (48.5)	5 (15.2)
Non-Compliant (n=217)	12.2 (7.6)	16 (7.4)	8 (3.7)	112 (51.6)	175 (80.6)	61 (28.1)
Trach Closed						
Compliant (n=138)	12.1 (5.9)	10 (7.2)	6 (4.3)	79 (57.2)	112 (81.1)	38 (27.5)
Non-Compliant (n=21)	15.6 (12.8)	1 (4.8)	1 (4.8)	15 (71.4)	16 (76.2)	6 (28.6)
Mobilization Day of Surgery						
Compliant (n=24)	8.0 (5.4)	1 (4.2)	2 (8.3)	10 (41.7)	12 (50.0)	4 (16.7)
Non-Compliant (n=212)	11.9 (7.7)	15 (7.1)	9 (4.2)	105 (49.5)	168 (79.2)	59 (27.8)
Early Mobilization						
Compliant (n=45)	9.0 (5.1)	3 (6.7)	3 (6.7)	17 (37.8)	27 (60.0)	9 (20.0)
Non-Compliant (n=212)	11.9 (7.7)	15 (7.1)	9 (4.2)	105 (49.5)	168 (79.2)	59 (27.8)

CI, Confidence Interval; SE, standard error; LOS, Length of Stay; ED, Emergency Department; ICU, Intensive Care Unit

© 2025 Wagoner CW et al. *JAMA Otolaryngology–Head & Neck Surgery*.

eTable 4. Unadjusted and Adjusted Model Results

Post-Operative Outcomes	Estimate or Odds Ratio (CI)	
	Unadjusted	Adjusted[†]
Post-operative Hospital Length of Stay (Days; n=251)	-0.71 (-1.34 – -0.08)[*]	-0.68 (-1.32 – -0.06)[*]
Readmitted to Hospital (Yes/No; n=257)	0.86 (0.65 – 1.23)	0.85 (0.62 – 1.19)
Visited ED (Yes/No; n=257)	0.90 (0.62 – 1.35)	0.90 (0.61 – 1.38)
Readmitted to ICU (Yes/No; n=257)	0.88 (0.74 – 1.04)	0.90 (0.75 – 1.07)
Experienced Any Complication (Yes/No; n=257)	0.72 (0.56 – 0.90)[**]	0.72 (0.56 – 0.90)[**]
Experienced Severe complications (Yes/No; n=257)	1.03 (0.86 – 1.22)	1.03 (0.87 – 1.23)

CI, Confidence Interval; SE, standard error; ED, Emergency Department; ICU, Intensive Care Unit

[*], $p < 0.05$; [**], $p < 0.01$

[†] = models adjusted for ‘age’, ‘sex’, and ‘operative risk (ASA)’

© 2025 Wagoner CW et al. *JAMA Otolaryngology–Head & Neck Surgery*.
