# Supplementary material for: Enhanced Recovery After Surgery Compliance and Outcomes for Head and Neck Reconstructive Surgery
Source: JAMA Otolaryngol Head Neck Surg. 2025 Feb 27;151(4):371–8. doi: 10.1001/jamaoto.2024.5393 (PMC11869090; doi:10.1001/jamaoto.2024.5393)
Supplement: Supplement 2. — Data sharing statement [file jamaotolaryngolheadnecksurg-e245393-s002.pdf]

Data Sharing Statement

Wagoner. Enhanced Recovery After Surgery Compliance and Outcomes for Head and Neck Reconstructive Surgery. *JAMA Otolaryngol Head Neck Surg*. Published February 27, 2025. doi:10.1001/jamaoto.2024.5393

Data
Data available: No
